# Genomic Profiling Reveals Differences in Primary Central Nervous System Lymphoma and Large B-Cell Lymphoma, With Subtyping Suggesting Sensitivity to BTK Inhibition

**DOI:** 10.1093/oncolo/oyac190

**Published:** 2023-01-18

**Authors:** Eric A Severson, James Haberberger, Amanda Hemmerich, Richard S P Huang, Claire Edgerly, Kelsie Schiavone, Adib Najafian, Matthew Hiemenz, Mirna Lechpammer, Jo-Anne Vergilio, Glenn Lesser, Roy Strowd, Julia Elvin, Jeffrey S Ross, Priti Hegde, Brian Alexander, Samuel Singer, Shakti Ramkissoon

**Affiliations:** Foundation Medicine, Morrisville, NC, USA; Foundation Medicine, Morrisville, NC, USA; Foundation Medicine, Morrisville, NC, USA; Foundation Medicine, Morrisville, NC, USA; Foundation Medicine, Morrisville, NC, USA; Foundation Medicine, Morrisville, NC, USA; Foundation Medicine, Morrisville, NC, USA; Foundation Medicine, Cambridge, MA, USA; Foundation Medicine, Cambridge, MA, USA; Foundation Medicine, Cambridge, MA, USA; Wake Forest Baptist Comprehensive Cancer Center, Wake Forest School of Medicine, Winston-Salem, NC, USA; Wake Forest Baptist Comprehensive Cancer Center, Wake Forest School of Medicine, Winston-Salem, NC, USA; Foundation Medicine, Cambridge, MA, USA; Foundation Medicine, Cambridge, MA, USA; Foundation Medicine, Cambridge, MA, USA; Foundation Medicine, Cambridge, MA, USA; Hackensack University Medical Center, Hackensack, NJ, USA; Foundation Medicine, Morrisville, NC, USA; Wake Forest Baptist Comprehensive Cancer Center, Wake Forest School of Medicine, Winston-Salem, NC, USA

**Keywords:** primary CNS lymphoma, DLCBL molecular subtyping, comprehensive genomic profiling, immune checkpoint inhibition

## Abstract

**Background:**

B-cell primary central nervous system (CNS) lymphoma (PCL) is diffuse large B-cell lymphoma (DLBCL) confined to the CNS. Less than 50% of patients with PCL achieve complete remission with current therapies. We describe the findings from comprehensive genomic profiling (CGP) of a cohort of 69 patients with PCL, 36 cases of secondary CNS lymphoma (SCL), and 969 cases of DLBCL to highlight their differences and characterize the PCL cohort. In addition, we highlight the differences in frequency of germinal center B-cell like (GCB) and non-GCB subtypes and molecular subtypes, particularly MCD and EZH subtypes, between PCL and DLBCL.

**Materials and Methods:**

Sixty-nine cases of B-cell PCL, 36 cases of secondary CNS lymphoma (SCL), and 969 cases of DLBCL were evaluated by CGP of 405 genes via DNAseq and 265 genes via RNAseq for fusions (FoundationOne Heme). Tumor mutational burden (TMB) was calculated from 1.23 Mb of sequenced DNA.

**Results:**

Genomic alterations with significant differences between PCL and DLBCL included *MYD88*, *ETV6*, *PIM1*, *PRDM1*, *CXCR4*, *TP53*, and *CREBBP*, while only *MYD88* was significantly different between SCL and DLBCL. PCL cases were significantly enriched for the MCD molecular subtypes, which have an excellent response to BTKi. We report a patient with a durable complete response to BTKi consistent with their genomic profile. EBV status, *CD274* amplification, and TMB status suggest that 38% of PCL patients may benefit from ICPI; however further study is warranted.

**Conclusion:**

CGP of PCLs reveals biomarkers, genomic alterations, and molecular classifications predictive of BTKi efficacy and potential ICPI efficacy. Given the limitations of standard of care for PCL, CGP is critical to identify potential therapeutic approaches for patients in this rare form of lymphoma.

## Teaser

This article reports on the genomic landscape and prevalence of immunotherapy biomarkers, including tumor mutational burden, in a large cohort of patients with lymphoma. This is the second largest cohort of B-cell primary central nervous system lymphoma (PCL) cases to date and the largest to compare the molecular subtypes between PCL, secondary central nervous system lymphoma, and diffuse large B-cell lymphoma.

**Implications for Practice**

Primary CNS lymphoma (PCL) is genomically distinct DLBCL and has a poor prognosis with standard therapies. For newer treatments, comprehensive genomic profiling (CGP) is critical, as it reveals molecular classifications, specifically MCD, that are predictive of BTKi efficacy, and biomarkers that may be predictive of immunotherapy efficacy (TMB-high, *CD274* amplification). Given the limitations of standard of care for PCL, CGP can direct patients to newer therapeutic approaches in this rare form of lymphoma.

## Introduction

Diffuse large B-cell lymphoma (DLBCL) is the most prevalent type of non-Hodgkin lymphoma (NHL), with over 20 000 new cases diagnosed in the US annually.^1^ A small subset of DLBCL cases arise within and are restricted to the central nervous system. These are termed primary CNS lymphoma (PCL), which has an annual incidence of 1400 cases in the US^2^ and accounts for 3% of all primary CNS tumors.^3^ In contrast, approximately 6% of systemic DLBCL cases^4^ will involve the CNS, which is termed secondary CNS lymphoma (SCL).

Gene expression profiling of DLBCL reveals 2 main molecular subtypes: germinal center B-cell-like (GCB) and activated B-cell-like (ABC or non-GCB). The genomic landscape of non-GCB DLBCL has been well characterized, with oncogenic driver mutations typically affecting NF-kB and B-cell receptor (BCR) activation.^5,6^ Conversely, GCB DLBCL often involves constitutive activation of BCL2, preventing apoptosis.^5^ The differences between the 2 groups have important prognostic implications, as non-GCB DLBCL is a predictor of poor response to R-CHOP.^7^ Previous studies have demonstrated that most PCL cases are of the non-GCB subtype.^8^

Further subclassification of DLBCL is now possible using genomic profiles using the LymphGEN tool.^9^ The most common molecular subtypes are MCD and EZB, which most typically correlate with non-GCB and GCB subtyping respectively. These subtypes are of particular importance as they can predict the outcome of various therapeutic approaches. For example, systemic DLBCL of the MCD subtype have a robust response to BTKi,^16^ with 100% 5-year event free survival (PFS) in younger patients when treated with BTKi plus R-CHOP versus <50% with R-CHOP alone).

Sequencing of CNS lymphomas in other cohorts has revealed a consistent spectrum of genomic alterations, specifically highlighting recurrent genomic alterations in the NF-kB pathway (*CD79B*, *MYD88*, *TNFAIP3*, and *CARD11*), with additional alterations such as loss of *CDKN2A*, *PRDM1*, and *IgH-BCL6* translocations.^10-16^

Standard-of-care therapy for CNS lymphomas involves chemotherapy (combination methotrexate and R-CHOP).^17-19^ In general these therapies have limited success, with a median progression-free survival (PFS) of 39 months with a 79% survival rate at 2 years.^20^ Historically, CHOP chemotherapy was standard treatment for DLBCL.^21^ The addition of rituximab was beneficial to DLBCL outcomes, but those improvements were muted in patients with PCL.^22^ Even for patients with PCL who achieve a complete response (CR), most do not have durable responses, with approximately 50% of patients experiencing refractory/relapsed disease.^23,24^ The variable outcomes for PCL patients with standard-of-care therapy highlight the need for improved treatment modalities. Promising new therapeutic modalities being explored for PCL clinical management include Bruton tyrosine kinase (BTK) inhibitors (BTKi) and immune checkpoint inhibitors (ICPI)/immunotherapy.

Ibrutinib, a BTKi, has performed well in DLBCL clinical trials, particularly in non-GCB patients compared with patients with GCB.^31,32^ This preferential effect is a result of the chronic activation of B-cell receptors in non-GCB cells,^6^ which ibrutinib targets. Notably, clinical trials of monotherapy ibrutinib in salvage treatment of PCL showed high overall (77%, 10/13) and complete (39%, 5/13) response rates.^33^ However, resistance to BTKi monotherapy may be conferred to patients with PCL harboring certain mutations. *MYD88*, notably *MYD88* L265P, has been shown to confer resistance to BTKi in non-GCB DLBCL; combination therapy with histone deacetylation inhibitors has shown some effectiveness in patients with *MYD88* genomic alterations.^34^*CARD11* mutations in follicular lymphoma confer BTKi resistance.^35^ Additionally, investigation into *CD79B* overexpression revealed induced BTKi resistance through AKT/MAPK pathways in DLBCL.^36^ Specifically in patients with the MCD and N1 molecular subtypes of non-GCB DLBCL, BTKi in conjunction with R-CHOP has resulted in significantly improved survival over R-CHOP alone, with a 3-year event free survival of 100% versus <50% for R-CHOP alone.^16^

Immunotherapy is a strong candidate across multiple tumor types. Mouse models of immunocompetent PCL have shown increased immune-factor infiltration and improvements in overall survival with ICPI.^25^ Moreover, immunohistochemistry of patients with PCL indicate 71% (12/17) of cases score positively for PD-L1, suggesting that ICPI may have value in this patient population.^26^ PCL is enriched for 9p24.1 amplification^27^ which increases PD-L1 expression; and PCL often has PD-L1 expression (59% of cases (*n* = 71) in one study^28^). These both provide an explanation for the increased ICP susceptibility.^29^ The use of nivolumab, in one cohort, showed complete responses in 80% (4/5) and 17-month PFS in 60% (3/5) of patients.^30^

This study describes the genomic landscape and prevalence of immunotherapy biomarkers, including tumor mutational burden (TMB), in a large cohort of PCL, SCL, and DLBCL patients. GCB and non-GCB status is compared as are the molecular DLBCL subtypes. This is the second largest cohort of PCL cases to date (*n* = 69), and the largest to compare the molecular subtypes between PCL, SCL, and DLBCL. The purpose is to demonstrate the utility of comprehensive genomic profiling (CGP) for identifying therapies for PCL patients that may be alternatives to standard approaches currently available. The use of CGP for identifying treatment strategies is highlighted by a case study of complete response to ibrutinib in a patient with refractory PCL.

## Methods

CGP was performed in a College of American Pathologists (CAP)-accredited, Clinical Laboratory Improvement Amendments (CLIA)-certified, New York State-regulated reference laboratory (Foundation Medicine, Inc.). This study was approved by the Western Institutional Review Board (IRB# 20152817) and includes a waiver of informed consent and a HIPAA waiver of authorization. All samples underwent central histopathologic review by a board-certified pathologist. DNA and RNA were extracted from formalin-fixed paraffin-embedded tumor samples, and next-generation sequencing was performed on hybridization-captured, adaptor ligation-based libraries to high, uniform coverage (>500×) for all coding exons of 406 cancer-related genes using DNAseq, and RNAseq in 265 genes (FoundationOne Heme assay). Sequence data were analyzed for clinically relevant classes of genomic alterations, including base pair substitutions, insertions/deletions, copy number alterations, and rearrangements/fusions. TMB was calculated on up to 1.2 Mb as the number of somatic, coding point mutations and indels per Mb of genome (low: <6; intermediate: 6-19; high: ≥20 mutations/Mb). Microsatellite instability (MSI) status was determined for each tumor and reported as MSI-Stable, MSI-Ambiguous and MSI-High.

CGP was performed on 69 cases of B-cell PCL, 36 cases of SCL, and 969 cases of DLBCL were evaluated for single nucleotide variants using the FoundationOne Heme assay^37^ during routine clinical care. The DLBCL cases were selected based on the disease classification assigned at the time of processing. The DLBCL, PCL, and SCL cases were confirmed by review of the provided supporting documentation (pathology reports) at the time of testing. Genomic alteration frequency comparison was performed using a Fisher’s exact test. TMB comparison was performed using an ANOVA Test (*P* < .05).

GCB and non-CGB classifications were assigned based on Hans algorithm^38^ using IHC results from pathology reports received at the time of CGP testing. All PCL and SCL cases were classified if IHC results were available and 100 random systemic DLBCL cases with IHC results were classified.

The LymphGEN tool (accessed at https://llmpp.nih.gov/lymphgen/index.php on 07April 2022) was used to assign DLBCL genetic subtypes to all samples.^39^

## Results

### Durable and Complete Response to BTK Inhibition in Refractory Primary CNS Lymphoma

**Figure 1.**
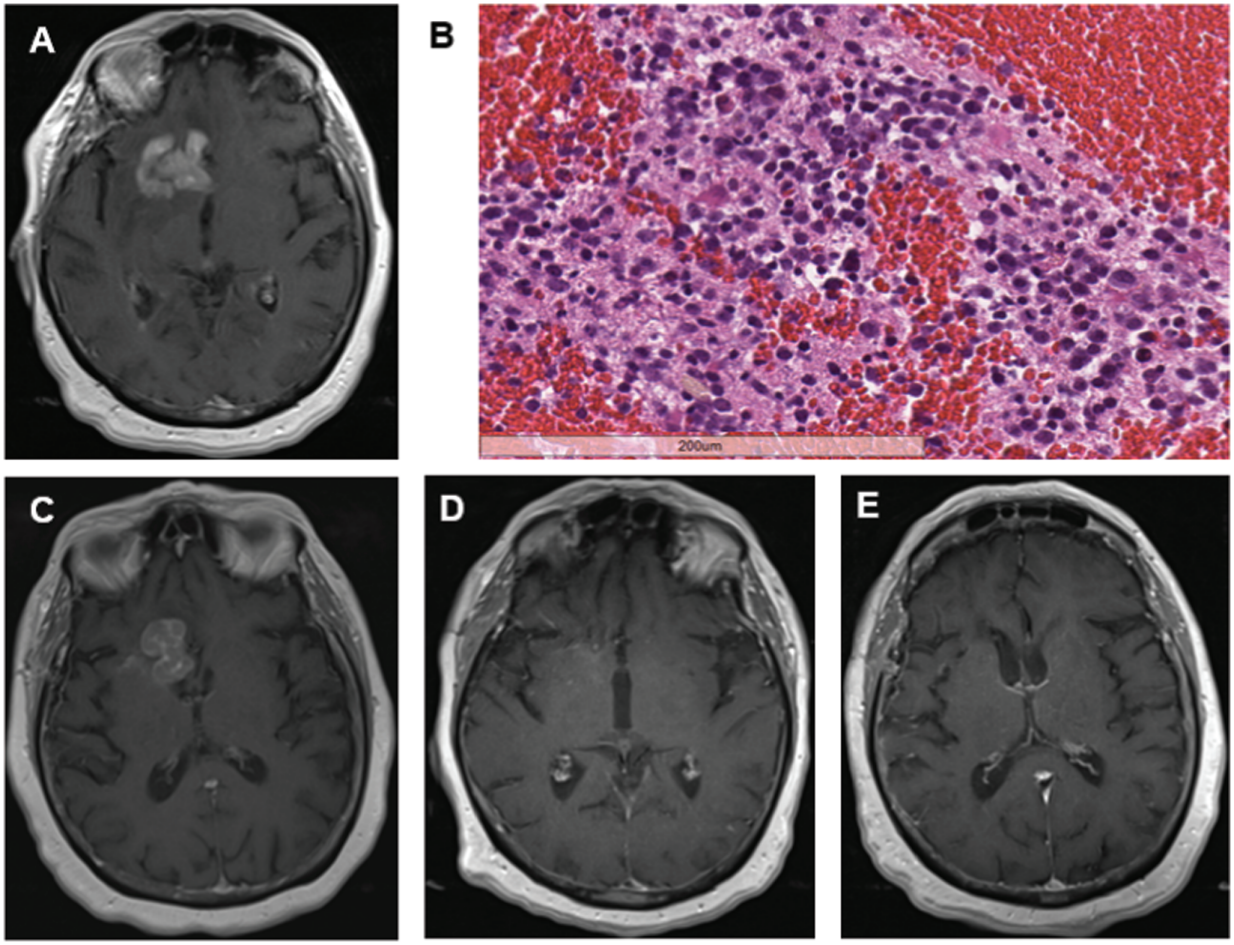
Patient with durable response to BTK inhibition in refractory PCL. (**A**) MRI (T1 + contrast) at diagnosis. (**B**) H&E of diagnostic material. (**C**) MRI after progression on methotrexate. (**D**) MRI after 8 cycles of ibrutinib showing complete response. (**E**) MRI showing durable response 5 months post-ibrutinib treatment.

A 79-year-old female presented with progressive lethargy, confusion, diabetes insipidus, and right sixth nerve palsy. A magnetic resonance imaging (MRI) study with contrast of the brain (Fig. 1A) revealed a right inferior frontal enhancing lesion. A biopsy was performed which revealed a densely cellular lesion comprised of a monotonous population of malignant lymphoid cells, consistent with the diagnosis of PCL (Fig. 1B). IHC studies revealed that the tumor cells expressed CD20, BCL2, and BCL6. Systemic evaluation including bone marrow biopsy was negative and CGP was performed on the diagnostic brain biopsy.

CGP of PCL tissues revealed the tumor to be wildtype for *CD79B*, *MYD88*, *CARD11*, and *TNFAIP3*, with genomic alterations detected in *SMO*, *CIITA*, *ETS1*, *HST1H1D*, and *SOCS1* (Supplementary Table S1). The tumor was found to be hypermutated with a tumor mutational burden of 28 mutations/Mb and was microsatellite stable.

The patient was treated with rituximab, methotrexate (3 g/m^2^), and temozolomide (100 mg/m^2^) on a 5/28 schedule with twice weekly intraommaya rituximab (25 mg) but progressed after the seventh cycle (4 months) (Fig. 1C). As a secondary line of treatment, ibrutinib (560 mg daily) and rituximab were initiated, with complete response noted after 5 months (Fig. 1D). She completed 12 months of ibrutinib, with rituximab discontinued due to cytopenias. Since completion of ibrutinib treatment, she has been followed for >5 years with observation only and remains clinically stable, asymptomatic and without evidence of recurrent enhancement on neuroimaging studies (Fig. 1E). A complete response to ibrutinib is consistent with prior reports for patients lacking pathogenic alterations in *CD79B*, *MYD88*, *CARD11*, and *TNFAIP3.*^33^

### Genomic Landscape of Primary CNS Lymphoma, Secondary CNS Lymphoma, and Systemic Diffuse Large B-Cell Lymphoma

The PCL cohort (*n* = 69) was 48% female and 52% male, aged 20-84 years (median 65 years). Our SCL cohort (*n* = 36) was 50% male and female, aged 10-87 years (median 63 years). Our DLBCL cohort (*n* = 969) was 37% female and 63% male, aged 0-88 years (median 62 years) (Table 1).

**Table 1.** Demographic description of patient cohort

Characteristics	Details
Primary CNS lymphoma (*n* = 69)	
Age range	20-84 years
Median age	65 years
Male	52%
Female	48%
Secondary CNS lymphoma (*n* = 36)	
Age range	10-87 years
Median age	63 years
Male	50%
Female	50%
Systemic diffuse large B-cell lymphoma (*n* = 969)	
Age range	0-88 years
Median age	62 years
Male	63%
Female	37%

In PCL, the 10 most frequently enriched genes were *MYD88* (40/69), *CDKN2A* (33/69), *CD79B* (20/69), *CDKN2B* (19/69), *PRDM1* (13/69), *PIM1* (13/69), *ETV6* (12/69), *MLL2* (10/69), *CARD11* (8/69), and *TP53* (8/69). In SCL, the 10 most frequently enriched genes were *MLL2* (12/36), *CDKN2A* (12/36), *TP53* (11/36), *MYD88* (10/36), *IGH*-*BCL2* (8/36), *BCL2* (7/36), *CDKN2B* (7/36), *PIM1* (6/36), *CD79B* (6/36), and *PRDM1* (5/36). In systemic DLBCL, the 10 most frequently enriched genes included *TP53* (348/969), *MLL2* (302/969), *IGH*-*BCL2* (256/969), *CDKN2A* (249/969), *CDKN2B* (165/969), *CREBBP* (164/969), *BCL2* (159/969), *TNFAIP3* (153/969), *MYD88* (150/969), and *TNFRSF14* (147/969) (Table 2).

**Table 2.** Frequency of genomic alterations in Primary CNS lymphoma versus Secondary CNS lymphoma vs Systemic diffuse large B-cell lymphoma

Gene	Primary CNS lymphoma (*n* = 69)		Secondary CNS lymphoma (*n* = 36)		Systemic diffuse large B-cell lymphoma (*n* = 969)		Primary vs secondary*	Primary vs systemic*	Secondary vs systemic*
	Number	Percentage	Number	Percentage	Number	Percentage			
*MYD88*	40	58.0%	10	27.8%	150	15.5%	**1.80E–02**	**9.90E–13**	1.60E–01
*CDKN2A*	33	47.8%	13	36.1%	256	26.4%	5.30E–01	**1.98E–03**	4.80E–01
*CD79B*	20	29.0%	6	16.7%	60	6.2%	4.80E–01	**8.54E–07**	9.00E–02
*CDKN2B*	19	27.5%	7	19.4%	169	17.4%	6.60E–01	1.40E–01	9.70E–01
*PRDM1*	14	20.3%	5	13.9%	41	4.2%	7.70E–01	**6.05E–05**	8.00E–02
*ETV6*	13	18.8%	4	11.1%	42	4.3%	5.90E–01	**2.28E–04**	1.90E–01
*PIM1*	13	18.8%	6	16.7%	68	7.0%	1.00E+00	**9.72E–03**	1.20E–01
*BCL6*	12	17.4%	9	25.0%	189	19.5%	6.30E–01	9.30E–01	5.90E–01
*MLL2*	10	14.5%	12	33.3%	304	31.4%	1.20E–01	**1.29E–02**	9.80E–01
*TP53*	8	11.6%	11	30.6%	349	36.0%	1.00E–01	**1.23E–04**	7.70E–01
*CARD11*	8	11.6%	1	2.8%	73	7.5%	3.50E–01	4.80E–01	6.90E–01
*B2M*	7	10.1%	4	11.1%	142	14.7%	1.00E+00	5.80E–01	9.70E–01
*CXCR4*	6	8.7%	0	0.0%	19	2.0%	2.10E–01	**1.86E–02**	1.00E+00
*BCL2*	4	5.8%	11	30.6%	308	31.8%	**1.00E–02**	**8.79E–06**	1.00E+00
*CD274*	4	5.8%	1	2.8%	29	3.0%	8.30E–01	5.00E–01	1.00E+00
*PDCD1LG2*	4	5.8%	2	5.6%	32	3.3%	1.00E+00	5.30E–01	5.50E–01
*CREBBP*	4	5.8%	6	16.7%	173	17.9%	2.10E–01	**2.88E–02**	1.00E+00
*TNFRSF14*	2	2.9%	3	8.3%	148	15.3%	5.50E–01	1.20E–02	5.50E–01

Bold text indicates significant comparisons at *P* < .05.

*P values for comparisons are corrected for multiple hypothesis testing.

**Figure 2.**
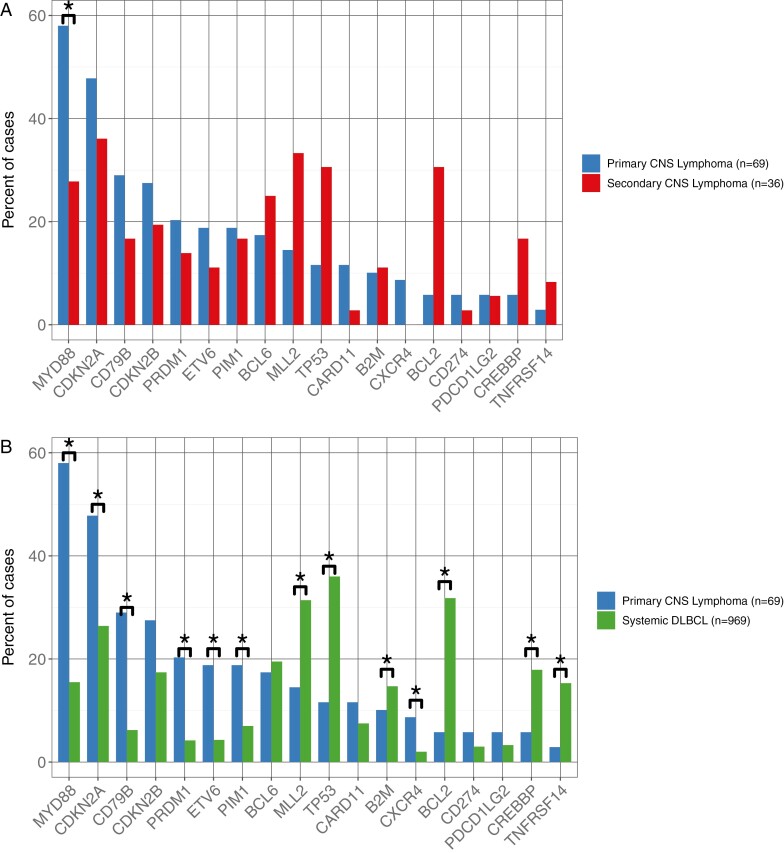
Genomic landscape in PCL, SCL, and systemic DLBCL. (**A**) Comparison of frequencies of genomic alterations in PCL cases (blue) compared to SCL cases (red). * = *P* < .05 by Fisher two tailed exact test corrected for multiple hypothesis testing. (**B**) Comparison of frequencies of genomic alterations in PCL cases (blue) compared to systemic DLBCL cases (green). * = *P* < .05 by Fisher two tailed exact test corrected for multiple hypothesis testing.

We performed a comparative analysis of genomic alterations detected in PCL versus Systemic DLBCL and found significant enrichment for genomic alterations (GAs) in the following genes: *MYD88* (58.0% vs 15.5%, *P* < .001), *CDKN2A* (47.8% vs 26.4% *P* ≤ .002), *CD79B* (29.0% vs 6.2%, *P* < .001), *ETV6* (18.8% vs 4.3%, *P* < .001), *PRDM1* (20.3% vs 4.2%, *P* < .001), *PIM1* (18.8% vs 7.0%, *P* ≤ .010), and *CXCR4* (8.7% vs 2.0%, *P* ≤ .019). GAs with significant enrichment in systemic DLBCL included *MLL2* (14.5% vs 31.4%, *P* ≤ .012), *TP53* (11.6% vs 30.6%, *P* < .001), *BCL2* (5.8% vs 31.8%, *P* < .001), *CREBBP* (5.8% and 17.9%, *P* ≤ .029), and *TNFRSF14* (2.9% and 15.3%, *P* ≤ .012) (Fig. 2a and 2b).

In comparing PCL and SCL, *MYD88* was significantly enriched in PCL (58.0% vs 27.8%, *P* = .018) and BCL2 was significantly enriched in SCL (5.8% vs 30.6%, *P* = .007). GAs conferring resistance to BTKi were seen more frequently in PCL than in SCL, such as *CARD11* (11.6% vs 2.8%, *P* = .346) and *CD79B* (29% vs 16.7%, *P* = .479); however, these differences were not statistically significant. None of the genes tested were found to be significantly different between SCL and systemic DLBCL (Table 2). These findings are consistent with and build upon previous reports of genomics in PCL and DLBCL (reviewer citations 1-6).^25,30-32^

### Co-occurring Alterations in PCL in the B-Cell Receptor Signaling Pathway

In this cohort, 68.1% (47/69) of patients with cumulatively had GAs in the B-cell receptor signaling pathway genes: *MYD88* (*n* = 40), *CD79B* (*n* = 20), *CARD11* (*n* = 8), and/or homozygous loss of *TNFAIP3* (*n* = 5). Enrichment in SCL and DLBCL for GAs in B-cell receptor signaling pathway *CD79B* (SCL: 16.7%, Systemic: 6.2%), *CARD11* (SCL: 2.8%, Systemic: 7.5%), *TNFAIP3* (SCL: 2.8%, Systemic: 15.8%), and *MYD88* (SCL: 27.8%, Systemic: 15.5%) is reported here as well.

### Classification of PCL, SCL, and DLBCL Cases With Hans Algorithm

**Figure 3. (:**
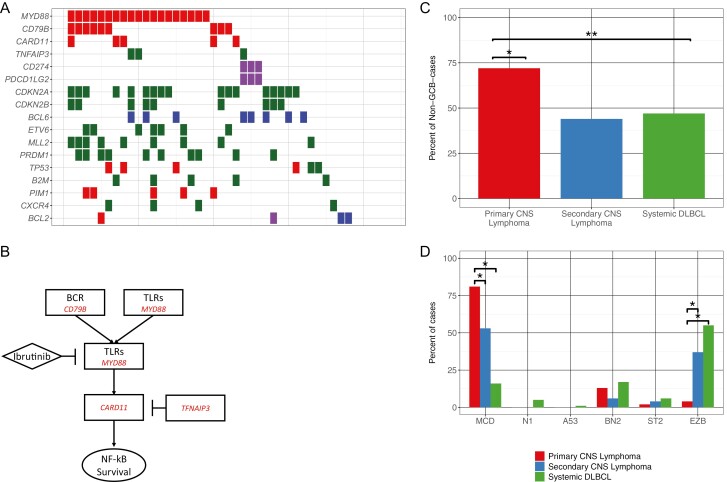
**A**) Co-mutation plot for primary CNS lymphoma cases. Red represents short-nucleotide variants, green represents truncations and homozygous deletions, purple represents copy number amplifications, and blue represents gene rearrangements. (**B**) Schematic of the B-cell receptor/nF-kB signaling axis and point of BTK inhibition. (**C**) Comparison of GCB vs non-GCB classification between PCL, SCL, and systemic DLBCL using Hans algorithm (* = *P* < .005, ** = *P* < .02). (**D**) LymphGEN classification by percent of cases in each class for PCL, SCL, and systemic DLBCL (* = *P* < .05).

As determined by Hans algorithm,^38^ significantly more PCL cases were of the non-GCB subtype compared with both the SCL (*P* < .02) and systemic DLBCL cases (*P* < .005). SCL and systemic DLBCL cases are not significantly different (P = .89). Seventy-two percent (36/50) of PCL samples were of the non-GCB subtype, 44% (11/25) of SCL samples were of the non-GCB subtype, and 47% (47/100) of systemic DLBCL samples evaluated were of the non-GCB subtype (Fig. 3C).

### Classification of PCL, SCL, and DLBCL Cases by LymphGEN

Of all DLBCL cases in our cohorts, 47% of cases were able to be assigned a classification based on genomic alterations identified and clinically reported using CGP results.

**Figure 4.**
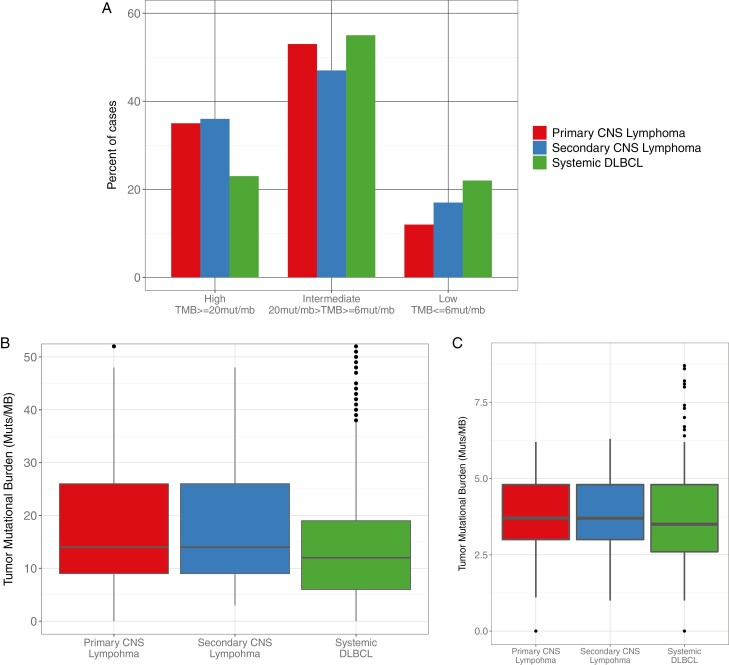
Comparison of tumor mutational burden between PCL, SCL, and systemic DLBCL. (**A**) Categorical comparison of TMB scores between PCL, SCL, and systemic DLBCL for TMB-high (≥20 mut/mb), TMB-intermediate (20 mut/mb > TMB > 6 mut/mb), and TMB-low (≤6 mut/mb). (**B**, **C**) Box and whisker plots comparing TMB for PCL, SCL, and Systemic DLBCL.

There were 23 (of 69) PCL cases, 14 (of 36) SCL cases and 448 (of 970) Systemic DLBCL cases with classifications determined by LymphGEN. Both PCL and SCL cases were significantly enriched for the MCD subtype as compared to systemic DLBCL (81.3% vs 15.9%, *P* < 2.5 × 10^-13^ and 52.6% vs 15.9%, *P* < .002 respectively). The most common subtype in systemic DLBCL was the EZB subtype at 55% of cases, with this subtype composing 4% of PCL cases (*P* < .1 vs systemic DLBCL) and 36.8% of SCL cases (*P* = .61 vs systemic DLBCL) (Fig. 4D). No other comparisons between molecular subtypes were statically significant.

Of the cases that had IHC results to perform a Hans classification, 30% (15 of 50) PCL cases, 44% (11 of 25) SCL cases, and 43% (43 of 100). Systemic DLBCL cases also were able to be classified by LymphGEN. Across all disease types, 59.6% (28 of 94) of non-GCB and 51.9% (42 of 81) of GCB cases had a classification assigned by LymphGEN. This difference is likely for 2 reasons. First, many MCD cases, which are predominantly non-GCB, were just below the confidence level for classification. If the confidence for calling MCD is lowered from a 0.5 probability to a 0.3 probability, an additional 32 cases are classified as MCD, which raises the percent of non-GCB cases with a classification to 36% (34 of 94). In addition, most A53 cases are of the non-GCB subtype, which are rarely called in the absence of chromosomal arm level data.

### Indicators of Potential Immunotherapy Benefit

Median (mean) TMB scores among PCL, SCL, and DLBCL were 14.0 mut/Mb (18.8 mut/Mb), 14.0 mut/Mb (21.1 mut/Mb), and 12.0 mut/Mb (17.5 mut/Mb), respectively. TMB-high patients, defined as >20 mut/Mb, comprised 34.8% (24/69), 33.3% (13/39), and 33.5% (233/969) of the PCL, SCL, and DLBCL cohorts. Neither the median TMB score, nor the percentage of cases presenting as TMB-high, were significantly different between the 3 groups.

Factors which may contribute to improved susceptibility to immunotherapies were explored. Four (5.8%) PCL cases were PD-L1 (*CD274*) amplified (range: 10-176 copies, median: 15 copies), with only one of those cases being TMB-high (Fig. 4A-C), and seven additional cases showed copy number gains at chromosome 9p (10.1%). In total, 11 (15.9%) cases showed some manner of amplification for CD274. For 32 samples, pathology reports included EBV status as determined by ISH. Of these 32 samples, 5 were positive for EBV (13.9%). In total, 37.8% of PCL cases (26/69) showed high TMB, chromosome 9p gains, and/or EBV positivity, which we considered indicators of potential benefit from immunotherapy.^40^

In SCL, *CD274* amplifications were found in a single patient (1/36, 2.8%) (Copy Number: 24). In systemic DLBCL, *CD274* amplifications were detected in 2.3% of patients (22/969) with copy numbers ranging from 6 to 179 copies, with a median of 13 copies.

## Discussion

CGP has the potential to suggest therapeutic strategies of value to patients, or to flag those likely to be ineffective. Given the variable outcomes for PCL patients, the ability to personalize treatment based on defined biomarkers is critical. In this study, we examined the genomic landscape of PCL, molecular subtyping of PCL, and the distribution of biomarkers such as TMB and explored how those results might be targeted by emerging therapeutic strategies.

Our survey of this patient population found that PCL cases were significantly more likely than SCL or systemic DLBCL samples to be of the non-GCB subtype (72% vs 44% and 47%, respectively)), as determined by reported IHC results using Hans algorithm, which is consistent with other studies characterizing PCLs^41^ and genomic alterations.

Molecular classification of DLBCL cases, whether PCL, SCL, or systemic DLBCL is valuable and avoids some of the pitfalls of the Hans classification. One difficulty with Hans classification, especially at a reference laboratory is the heterogeneity of IHC results as reported across different institutions. Data available from CGP only was sufficient to classify approximately 50% of cases. This is compared to 63% of cases that were able to be identified in the Wright et al study.^9^ Integration of arm level chromosomal gains and losses from karyotyping and additional FISH results unavailable in a reference lab setting likely account for the difference and could be integrated in clinical practice. In particular, the A53 subtype was rarely called (2 of 1058 cases) without chromosomal arm level data, compared to the expected rate of 6% in the presence of chromosomal arm level data, which accounts for at least half of that difference.

Of interest, while SCL and systemic DLBCL had similar classification results based on Hans classification, both PCL and SCL were significantly more likely to be of the MCD molecular subtype than systemic DLBCL, while systemic DLBCL was significantly more likely to be of the EZB subtype. Of note, all PCL cases that had both Hans classification were of the MCD subtype, while some of those cases were determined to be GCB subtype with Hans classification, which highlights the difficulties of using IHC results abstracted from pathology reports across many different institutions. Per Wright *et al* nearly all cases classified as MCD should be of the non-GCB subtype.

Of the cases that had IHC results to perform a Hans classification, 30% of non-GCB cases could be classified compared to 52% of GCB cases. This difference is likely for 2 reasons. First, many MCD cases, which are predominantly non-GCB, were just below the confidence level for classification. If the confidence for calling MCD is lowered from a 0.5 probability to a 0.3 probability, an additional 32 cases are classified as MCD, which raises the percent of non-GCB cases with a classification to 36% (34 of 94). In addition, most A53 cases are of the non-GCB subtype, which are rarely called in the absence of chromosomal arm level data.

Molecular subtyping of this kind has significant prognostic value, both for predicting overall patient outcomes but also for determining the potential responsiveness to different therapies. BKTi represents one such directed therapy, which is more efficacious in non-GCB DLBCLs such as PCL. This was specifically demonstrated for systemic DLBCL in Wright *et al* where excellent response of the MCD and N1 subgroups to BTKi was demonstrated.^9^ Systemic DLBCL of the subtypes MCD and N1 have a robust response to BTKi,^16^ with 100% 5-year event free survival (PFS) in younger patients when treated with BTKi plus R-CHOP versus <50% with R-CHOP alone). This is of particular importance in PCL, where there the MCD subtype is significantly enriched.

There are resistance mechanisms that allow tumors to bypass BTK inhibition. In a phase I clinical trial, BTK inhibition with ibrutinib resulted in a complete response in patients with wild-type *CD79B*, *CARD11*, and *TNFAIP3*, with partial or complete resistance associated with GAs in any of these B-cell receptor pathway genes.^33^*MYD88* L265P has been characterized in PCL as being associated with resistance to BTKi monotherapy.^34,42^ Overexpression of *CD79B* can induce BTKi resistance by enhancing AKT/MAPK function in DLBCL.^32,36^ One clinical trial in PCL using BTKi in PCL also implicated *CARD11* and *TNFAIP3*, being members of the NF-kB pathway, as potential means of resistance development.^33^ The results of clinical trials based on molecular subtypes for BTKi in systemic DLBCL merits caution in interpreting these earlier findings and indicates that specific studies of PCL and BTKi based on molecular subtyping should be performed.

In our PCL cohort, potential de novo resistance to BTK inhibition was detected in 68.1% (47/69) of samples, with alterations detected in *MYD88* (40/69), *CD79B* (20/69), *CARD11* (8/69), and *TNFAIP3* (5/69). *MYD88* mutations were found to be significantly more common in PCL than in either SCL (58% vs 28%, *P* = .02) or systemic DLBCL (58% vs 15%, *P* = 9.9 × 10^-13^). Similarly, *CD79B* was found to be significantly enriched in PCL compared to systemic DLBCL (29%, 6%, *P* = 8.5 × 10^-7^).

ICPIs, such as nivolumab or pembrolizumab, have also shown value as novel therapeutic strategies, particularly against oncogenic targets with immune-evading mutations. In one Hodgkin’s lymphoma cohort, patients showed an 87% (20/23) response rate to nivolumab.^43^ Similarly, patients with 9p24.1 amplifications with relapsed PCL all (5/5) showed clinical and radiographic responses, with most (3/5) showing disease-free progression after 17 months.^44^ In addition to general effectiveness, several genomic biomarkers are associated with the effectiveness of ICPIs. Clinical studies have suggested that TMB may be useful in predicting the outcome of immunotherapy when looking at non–small cell lung cancer^45^; however, not all tumor types benefit from immunotherapy with high TMB.^15^ Additionally, EBV+ cells of lymphoproliferative disorders have been shown to express PD-L1 in immunocompetent patients, which may indicate immunotherapy susceptibility.^46,47^

CGP of PCLs in this study revealed many samples harbored biomarkers indicative of potential immunotherapeutic susceptibility, including high TMB (24/69, 34.8%), *CD274* amplification (4/69, 5.8%), Chr9 or Chr9p gains (11/69, 10.1%), and/or EBV positivity (6/69, 8.7%), affecting 49.3% (34/69) of patients in total. These data indicate that these patients could potentially show enhanced responses to ICPIs. Further work should be done specifically in PCL to investigate the efficacy of immunotherapy in relation to these genomic biomarkers.

Primary CNS lymphoma (PCL) is a genomically distinct type of DLBCL and has a poor prognosis with standard therapies. For newer treatments, comprehensive genomic profiling (CGP) and molecular subtype assignment is critical, as it reveals biomarkers and classifications that may be predictive of immunotherapy efficacy (TMB-high, *CD274* amplification) and molecular classifications, specifically MCD and N1, that are predictive of BTKi efficacy. Given the limitations of standard of care for PCL, CGP can direct patients to newer therapeutic approaches in this rare form of lymphoma.

## Supplementary Material

oyac190_suppl_Supplementary_Material

## Data Availability

The data underlying this article will be shared on reasonable request to the corresponding author.
