# Supplementary material for: Genomic Profiling Reveals Differences in Primary Central Nervous System Lymphoma and Large B-Cell Lymphoma, With Subtyping Suggesting Sensitivity to BTK Inhibition
Source: Oncologist. 2023 Jan 18;28(1):e26–35. doi: 10.1093/oncolo/oyac190 (PMC9847534; doi:10.1093/oncolo/oyac190)
Supplement: oyac190_suppl_Supplementary_Material [file oyac190_suppl_supplementary_material.docx]

**Supplemental Table 1**

| **Gene** | **Alteration** |
| --- | --- |
| SMO | R199W |
| SOCS1 | A17T |
| CIITA | Q797* |
| ETS1 | Splice Site 82+1_82_6GTGAGT>CTGAGA |
| HIST1H1D | K179fs*15 |
| CDKN2A | Homozygous Loss |
| CDKN2B | Homozygous Loss |
| CD79B | Wildtype |
| MYD88 | Wildtype |
| CARD11 | Wildtype |
| TNFAIP3 | Wildtype |
| TMB Score | 27.7 |
| MSI Status | MSS |
